# Overexpression of the Chromosome Partitioning Gene *parA* in *Azorhizobium caulinodans* ORS571 Alters the Bacteroid Morphotype in *Sesbania rostrata* Stem Nodules

**DOI:** 10.3389/fmicb.2019.02422

**Published:** 2019-10-24

**Authors:** Hsiao-Lin Chien, Wan-Zhen Huang, Ming-Yen Tsai, Chiung-Hsiang Cheng, Chi-Te Liu

**Affiliations:** ^1^Institute of Biotechnology, National Taiwan University, Taipei, Taiwan; ^2^Institute of Molecular and Comparative Pathobiology, School of Veterinary Medicine, National Taiwan University, Taipei, Taiwan; ^3^Agricultural Biotechnology Research Center, Academia Sinica, Taipei, Taiwan

**Keywords:** chromosome partitioning, nitrogen fixation, bacteroid formation, nodule development, cell cycle, plant defense

## Abstract

*Azorhizobium caulinodans* ORS571 is a diazotroph that forms N_2_-fixing nodules on the roots and stems of the tropical legume *Sesbania rostrata*. Deletion of the *parA* gene of this bacterium results in cell cycle defects, pleiomorphic cell shape, and formation of immature stem nodules on its host plant. In this study, we constructed a *parA* overexpression mutant (P*nptII*-*parA*) to complement a previous study and provide new insights into bacteroid formation. We found that overproduction of ParA did not affect growth, cell morphology, chromosome partitioning, or vegetative nitrogen fixation in the free-living state. Under symbiosis, however, distinctive features, such as a single swollen bacteroid in one symbiosome, relatively narrow symbiosome space, and polyploid cells were observed. The morphotype of the P*nptII*-*parA* bacteroid is reminiscent of terminal differentiation in some IRLC indeterminate nodules, but *S. rostrata* is not thought to produce the NCR peptides that induce terminal differentiation in rhizobia. In addition, the transcript patterns of many symbiosis-related genes elicited by P*nptII*-*parA* were different from those elicited by the wild type. Accordingly, we propose that the particular symbiosome formation in P*nptII-parA* stem-nodules is due to cell cycle disruption caused by excess ParA protein in the symbiotic cells during nodulation.

## Introduction

Nodule formation in plants of the Fabaceae or Leguminosae family can be classified as determinate or indeterminate; the major difference between these types is the presence or absence of an active meristem in the fully developed organ (Hirsch, 1992; Sprent, 2007). During nodule development, rhizobia are released from infection threads, engulfed by plant-derived symbiosome membranes via endocytosis and maintained in the infected plant cells. Subsequently, the rhizobia undergo cellular differentiation to form nitrogen-fixing bacteroids (Tautz, 1992; Jones et al., 2007; Gibson et al., 2008; Downie, 2014). During the bacteroid formation process, not only cellular structure but also gene expression and metabolic activities are comprehensively changed to meet the needs of nitrogen fixation and adapt to the cellular environment of the nodule (Kereszt et al., 2011).

The morphotypes of the bacteroids within the nodules can be classified into swollen and non-swollen, which are mainly determined by the host legume clade rather than by the nodule type (Oono and Denison, 2010; Van De Velde et al., 2010; Kereszt et al., 2011). In some determinate nodules, such as those of *Glycine, Phaseolus, Vigna* and *Lotus*, the bacteroids are comparable in both shape and size to the corresponding free-living bacteria (i.e., the bacteroids are non-swollen). In other types of determinate nodules, such as those of *Arachis* and *Aeschynomene* (Dalbergoid clade legumes), the bacteroids are swollen. In some indeterminate nodules, such as those of *Cicer* and *Glycyrrhiza*, which belong to Inverted Repeat Lacking Clade (IRLC), non-swollen bacteroids are formed(Montiel et al., 2016). In contrast, in other IRLC legume, rhizobia form highly elongated or branched cells (i.e., swollen bacteroids) in indeterminate nodules, such as those of *Medicago, Pisum*, *Trifolium* or *Vicia*. The features of such bacteroids are cell enlargement, genome amplification (endoreduplication) and membrane permeabilization (Mergaert et al., 2006). Because these polyploid bacteroids lose their ability to resume growth, this type of cellular process is also called terminal bacteroid differentiation (TBD); the resulting bacteroids are proposed to be more effective in nitrogen fixation than the reversibly differentiated, reproductive bacteroids (Oono and Denison, 2010). It has been considered that host legumes have developed several strategies to control and dominate their endosymbiotic rhizobia. TBD is now known to be triggered by plant factors that show similarities to defensin-like innate immunity factors, designated as nodule-specific cysteine-rich (NCR) peptides (Mergaert et al., 2006; Wang et al., 2010).

*Sesbania rostrata* is a semiaquatic annual legume that originates from West Africa. It has been classified as a member of the Papilionoid subfamily and Robinioid clade (Sprent, 2007). *S. rostrata* can form nitrogen-fixing nodules at both the adventitious root primordia (stem nodules) and the bases of lateral roots (root nodules) with its microsymbiont *Azorhizobium caulinodans* (Ndoye et al., 1994). The mature stem or root nodules of *S. rostrata* are considered to be of the determinate type. However, the nature of nodule development is heterogeneous, and the early stages in *S. rostrata* also resemble those of indeterminate nodules, such as the process of proximal-distal differentiation and the place of origin (middle-inner cortex) (Ndoye et al., 1994; Goormachtig et al., 1997).

In bacteria, the process of chromosome partitioning involves the separation and positioning of daughter chromosomes in each cell cycle (Hiraga, 1993). Accurate distribution of the daughter chromosomes at cell division is essential to ensure that each cell receives a complete copy of the genome (Gordon and Wright, 2000). The best characterized family of genes that play a specific role in chromosome segregation is referred to as the *parAB* family, members of which encode ParA and ParB proteins (Williams and Thomas, 1992; Hiraga, 1993; Ireton et al., 1994; Gordon et al., 1997; Sharpe and Errington, 1998; Hiraga, 2000; Bignell and Thomas, 2001). This process is very efficient and precise, and cells lacking chromosomes are very rarely produced (Hiraga, 2000). Deletion or overexpression of *par* genes affects chromosome partitioning in many bacteria, such as *Bacillus subtilis*, *Caulobacter crescentus*, *Pseudomonas aeruginosa*, and *Myxococcus xanthus*, resulting in accumulation of anucleate cells, late cell growth and abnormal cell morphology (Easter and Gober, 2002; Ogura et al., 2003; Lee and Grossman, 2006; Lasocki et al., 2007; Kusiak et al., 2011; Mierzejewska and Jagura-Burdzy, 2012; Bartosik et al., 2014; Iniesta, 2014). In addition, mutations in the *par* system mutations affect DNA replication (Murray and Errington, 2008), cytokinesis (Mohl et al., 2001), sporulation (Ireton et al., 1994), and motility (Lasocki et al., 2007; Bartosik et al., 2009).

In our previous study, we found that the null mutant of a chromosome partitioning gene (*parA*) (genomelocus tag AZC_4711 [accession number AP009384], region 5360130.0.5360978 [DDBJ/EMBL/GenBank databases]) of *A. caulinodans* ORS571 (strain ORS571-Δ*parA*, designated Δ*parA*) had an altered cell cycle and formed elongated or branched cells with higher nucleic acid contents (polyploidy) (Liu et al., 2011). We assumed that the Δ*parA* cells had already differentiated prior to invading their host plant. When *S. rostrata* was inoculated with Δ*parA*, immature stem nodules with varying degrees of maturity were generated. The transcript level of the *parA* was inversely correlated with the maturity of the nodule, and the transcript was absent in fully mature bacteroids. Accordingly, we propose that the *parA* gene not only plays a crucial role in the partitioning of chromosomes but also participates in the bacteroid formation process in *S. rostrata* stem nodules.

Despite many investigations of the roles of the ParAB proteins in various bacteria, very little is known about how the chromosome partitioning system is involved in bacteroid development and nitrogen fixation during rhizobium-legume symbiosis. The aim of this study was to elucidate the effects of ParA overproduction in *A. caulinodans* ORS571 under free-living conditions and during symbiosis to complement the previously reported studies of the *parA* null mutant. Thus, we constructed a P*nptII*-*parA* mutant strain harboring a plasmid expressing *parA* from a strong constitutive promoter (P*nptII*), to analyze the cell morphology, viability and symbiotic features of *A. caulinodans* ORS571 under *parA* overexpression.

## Materials and Methods

### Biological Materials

The bacterial strains and plasmids used in this study are listed in Table 1. Derivatives of *A. caulinodans* strain ORS571 (Dreyfus et al., 1988) were grown at 37°C in TY medium (Beringer, 1974) or L2-N medium with appropriate antibiotics. L2-N medium is a synthetic nitrogen-deficient medium modified from LO medium (Dreyfus et al., 1983). *Escherichia coli* strains were grown in LB broth at 37°C. Antibiotics were used when appropriate at the following concentrations: nalidixic acid 25 μg/ml, kanamycin 50 μg/ml, ampicillin 100 μg/ml, and tetracycline 20 μg/ml.

**TABLE 1:** Bacterial strains and plasmids used in this study.

**Strains and plasmids**	**Description or relevant phenotype^a^**	**References**
***Escherichia coli***		
DH5α	*endA1 hsdR17 supE44 thi-1 recA1 gyrA96 relA1* Δ (*argF*-*lacZYA*)*U169*φ 80*lacZ* ΔM15	Invitrogen
S17-1	RP4 *tra* region, mobilizer strain, for conjugation, Sp^r^	Simon et al., 1983
***Azorhizobium caulinodans***		
ORS571	Wild-type, Nx^r^	Dreyfus et al., 1988
ORS571-Δ*parA*	Null mutation of a putative chromosome partitioning gene (*parA*), Nx^r^	Liu et al., 2011
ORS571-P*nptII*-*parA*	Constitutive expression of *parA* gene driven by a *nptII* promoter, Nx^r^, Ap^r^, Tc^r^	This study
**Plasmids**		
*nptII*:*parA*:pFAJ1708	pFAJ1708 with 940-bp *Bam*HI/*Kpn*I fragment, transcription of *parA* driven by a constitutive *nptII* promoter, Ap^r^, Tc^r^	This study
pFAJ1708	Broad-host-range plasmid containing *nptII* promoter, Ap^r^, Tc^r^	Dombrecht et al., 2001

**^*a*^*Abbreviations: Nx^*r*^, nalidixic acid 25 μg/ml; Km^*r*^, kanamycin 50 μg/ml; Ap^*r*^, ampicillin 100 μg/ml; Tc^*r*^, tetracycline 20 μg/ml.*

*S. rostrata* seeds were treated with concentrated sulfuric acid for 8 min and then placed under dripping water for an hour to induce rapid and uniform germination. Seedlings were grown for 3 weeks before inoculation at 35°C under a 24-h light regime by light-emitting diode (LED) illumination (Maxima 5000 SLD 5100, Neotroni, Taiwan) at an intensity of 30,000 lux (0.5 mmol photons m^–2^ s^–1^). Three-week-old plants were inoculated with the desired azorhizobial strains at the mid-exponential phase (∼5 × 10^8^ cells per ml) between the first and second stem internodes, where stem nodule development is synchronized (Donald et al., 1986). All developmental nodulation tests were performed in at least in triplicate.

### Construction of *parA* Overexpression Mutant

To construct a plasmid that expresses the *parA* gene constitutively under the control of the *nptII* promoter, the *parA* gene was amplified by PCR with the primers parA-F and parA-R, and the resulting DNA fragment (943 bp) was digested with *Bam*HI and *Kpn*I for cloning in the replicative broad-host-range plasmid pFAJ1708 (Dombrecht et al., 2001). The resulting plasmid was designated *nptII*-*parA*:pFAJ1708 (Supplementary Figure S1). All the PCR primer pairs used for plasmid construction are shown in Table 2. The *parA* sequence in the construct was verified by subsequent sequencing. The resulting plasmid was conjugated into *A. caulinodans* ORS571 via *E. coli* S17-1 (λ pir). The resulting strain, ORS571-P*nptII*-*parA* (hereafter abbreviated to P*nptII*-*parA*), was selected by tetracycline and ampicillin resistance.

**TABLE 2:** Primers used in this study.

**Gene name**	**Locus**	**Forward**	**Reverse**	**Product size (bp)**	**References**
**Construct *parA* overexpression mutant**					
parA-*Bam*HI-F^a^		GGGATCCCGACCGCGAAGGGGAAAAC		942	This study
parA-*Kpn*I-R^b^			GGGTACCCGTTCCTCGTTCCTTCACAG		This study
***Azorhizobium caulinodans* ORS571 quantitative RT-PCR**					
16S		ACGGATTTCTTCCAGCAATG	ACCGGCAGTCCCTTTAGAGT	130	Akiba et al., 2010
*nodD*	Azc_3792	AACCCCCGATCTGGGTAAT	CATCATTTGGGATGCATGG	64	This study
*noeC*	Azc_3810	ACTCGCCTCTCACCTTTCCT	ATCGTATGTCGCACTCTCGG	50	This study
*nodZ*	Azc_3811	CACAATTAGGTGATCATAGAACTCG	TTGCTGTCTCATGTGGTGCT	64	This study
*nodB*	Azc_3817	GAGCGCCGCTAATGTCTG	CCAGATGAAGCTGCGATG	60	This study
*nolK*	Azc_3850	ATCGCATCTTCTGCCTGC	CTCACACTTTCGCTACCACA	69	This study
*nifD*	Azc_1040	CGCACATCGCCAACACCA	ACCGTCCGCCAGATAGGC	68	This study
*nifH2*	Azc_1041	GACCTGGCTCTCGTCCAC	CACTATCGCAACCTCGCTGA	135	This study
*nifA*	Azc_1049	CCTTCTCATGGCCGAACA	CCTTCGTGAAGGTGAACTGC	72	This study
*fixA*	Azc_3447	ATGCGTCAGGGTGTGCCC	CGCCGAAGGTGAGTGCCT	157	This study
*fixN*	Azc_4523	CATCACGCAGGGCAAGGAA	CCGAGGAAGACAAGGAAATAGGT	62	This study
*expA4*	Azc_3331	CCGAGGACTATGTGAACGAG	TGGCGATGGAGGTGGAAC	71	This study
*bacA*	Azc_4674	GAACTCGGTCAGTCCCTCG	CGTTTCCCGTGCCCTTCT	98	This study
*oac3*	Azc_1831	TGGACTTCGCCTGCTCCT	CCTGCGTATTTCCTCGCCC	95	This study
*oac2*	Azc_1832	GTGGAATGTCCGCTCGAA	TCACCACCGCCGAGTATC	146	This study
*oac1*	Azc_1833	AGGCGGAATAGGGCGAAT	CACCAACATCAACGGCAC	188	This study
*dnaA*	Azc_1047	TCGGCGGTCAGATACACC	CGTTGGCCTCGGTAAGAC	87	This study
*ftsZ*	Azc_4564	CGACGCCAACATCATCCTC	ATCTGCTCGGGAACCACC	108	This study
*parA*	Azc_4711	CCTCTCCATCCACGGCATC	CGCACATCCTCCACCACC	78	This study
*parB*	Azc_4712	CCAAGGTCATCGGCAAGAG	GGCAGTTTCAGCAGGCGG	61	This study
***Sesbania rostrata* quantitative RT-PCR**					
*Srubi*		GATTTTTGTGAAGACCTTGACGGG	CACAGACCCATTACACATCCACAAG	300	Corich et al., 1998
*SrPI1*		TGGCAATTCTTGTGCCTAGTG	TGCAATGCTCAAACCCAGA	134	Lievens et al., 2004
*Srprx1*		TTCTGGAGGACACACGATTG	TAGTAGTTGACTTTCCTGCAGTC	390	Den Herder et al., 2007
*SrGA20ox1*		AGAGCCGACGAAGATACCCT	GCCGTACAAAGTAGAATTAGGTTAAG	231	Lievens et al., 2005

*^*a*^The *Bam*HI site is underlined. ^*b*^The *Kpn*I site is underlined.*

### Western Blot Analysis

Mid-exponential-phase bacterial strains were harvested and then homogenized by French press (Avestin EmulsiFlex-C3, Canada) at 18,000 psi. The soluble proteins (10 μg) were analyzed with 12.5% SDS-PAGE and subsequently analyzed with Western blot (Towbin et al., 1979). The rabbit anti-AzoParA IgG (LTK BioLaboratories, Taiwan) was used as the primary antibody for ParA detection. The horseradish peroxidase (HRP)-conjugated goat anti-rabbit IgG (Millipore) was used as a secondary antibody. Signals were detected by Western HRP and AP chemiluminescent substrates (Millipore) and visualized by BioSpectrum 510 (UVP).

To quantify the endogenous ParA proteins in *A. caulinodans* derivatives, Western blot signals were measured using ImageJ as described previously (Schneider et al., 2012).

### Acetylene Reduction Assay of Free-Living Bacteria and Stem Nodules

The biological N_2_ fixation (BNF) ability of free-living azorhizobial derivatives and stem-nodules was determined by acetylene reduction assay (ARA) (Dilworth, 1966). To assess the BNF ability of free-living bacteria, azorhizobial cells were collected at mid-exponential phase in TY broth by centrifugation, and the cells were washed twice with L2-N medium and suspended in L2-N medium to an optical density at 600 nm (OD_600_) of 0.1. In the following incubation, 50 ml aliquots of culture were transferred into 250 ml Erlenmeyer flasks sealed with sterile rubber septa. The gas phase in the flask was replaced with N_2_ gas containing 15% air (∼3% O_2_) and 10% C_2_H_2_ and incubated at 37°C with shaking at 200 rpm. After incubation for 15 h, 0.5 ml gas samples were taken from the flask, and the ethylene concentration was assayed using a gas chromatograph (HITACHI, G-3000) equipped with a *HayeSep T80/100* packed column (Supelco) and a flame ionization detector (Suzuki et al., 2007). The OD_600_ of each sample was measured immediately following the gas sampling.

Ten stem-nodules were excised from individual plants and placed into 15-ml vials sealed with a butyl rubber septum. The air in the vials was replaced with 10% (vol/vol) C_2_H_2_, and the vials were incubated at 37°C for an hour. After incubation, 0.5 ml of gas was sampled from each vial, and the concentrations of acetylene and ethylene were measured.

### Optical and Electron Microscopic Analyses

For observation of free-living bacteria, each bacterial strain was grown in TY medium until the mid-exponential phase (around optical density at 600 nm [OD_600_] of 0.5). Broth cultures were stained with 4,6-diamidino-2-phenylindole (DAPI; Sigma-Aldrich, 28718-90-3) at 10 μg/ml for 5 min at 25°C according to the method reported by Rowe and Summers (1999). Cells in suspension were mounted and examined by light microscopy (BX51, Olympus, Japan) under a bright or fluorescent field by use of the U-MWU2 filter set with UV excitation (excitation spectrum, 330 to 385 nm; emission spectrum, 420 nm; Olympus, Japan).

Stem-nodules were harvested at 7 days post inoculation (dpi) and fixed in FAA solution (formalin: acetic acid: 50% alcohol = 1: 1: 18) overnight for microscopic analysis. FAA-fixed nodules were embedded in 5% (w/v) agar and sectioned using a microslicer (DTK-1000, Dosaka, EM, Japan). Sliced sections were stained with 0.05% toluidine blue O (TBO) (O’brien et al., 1964) and observed using light microscopy as described above.

For transmission electron microscopy (TEM) analysis, 7 dpi stem nodule samples were fixed in 2.5% (w/v) glutaraldehyde at 4°C and then shaken overnight. The fixed nodules were washed with 0.1 M sodium phosphate buffer (pH = 7.2), post-fixed in 1% osmium tetroxide (w/v), dehydrated in an ethanol series, and then embedded in Spurr’s resin (Electron Microscopy Sciences, United States) for conventional TEM as described previously (Spurr, 1969). Semithin sections (500 nm) and ultrathin sections (70 nm) were taken from the resin-embedded samples using a Reichert-Jung Ultracut E ultramicrotome (Reichert-Jung, United States). The ultrathin sections for conventional TEM were collected on Pioloform-coated copper grids and stained with uranyl acetate (10 min) and lead citrate (5 min) before being viewed with a JEOL JEM-1400 transmission electron microscope (JEOL Ltd., Japan).

### Bacteroid Isolation From *S. rostrata* Stem-Nodules

Bacteroids were isolated from 5 dpi stem nodules of *S. rostrata* by a method modified from that described by Tsukada et al. (2009). Stem-nodules (5 g) were homogenized with 0.5 g of polyvinylpyrrolidone (Sigma-Aldrich, 9003-39-8) and 2 ml of Mg-phosphate buffer (2.5 mM MgCl_2_, 50 mM potassium phosphate, pH 6.8) using a cold mortar and pestle. The paste was diluted with 10 ml ice-cold Mg-phosphate buffer and passed through a 40 μm filter (BD Biosciences) to collect the filtrate. The filtrate was centrifuged at 500 *g* for 2 min at 4°C to obtain the supernatant and remove the plant cell materials, and this step was repeated several times until no obvious green pellet remained. The supernatant was then centrifuged at 5,000 × *g* for 5 min at 4°C to collect the bacteroids.

### Flow Cytometric Analyses

Cultured bacteria and bacteroids were fixed in 90% ethanol overnight at −20°C. Cells were then washed twice with PBS followed by centrifugation for 2 min at 1,200 × *g*. Pelleted cells were stained with propidium iodide (PI)-RNase staining buffer solution (BD Biosciences, 550825) for 30 min at room temperature. For each flow cytometry experiment, the DNA content was measured in a population of 20,000 cells with a Cytomics FC500 analyzer (Beckman Coulter Ltd.). Data analysis was performed with CXP software (Beckman Coulter Ltd.) (Wake and Errington, 1995).

### RNA Isolation and Purification

Bacteria were grown in TY medium until the optical density of the culture at 600 nm [OD_600_] reached 0.5 or in L2 medium for 15 h (the detailed conditions are described in the ARA assay session) and then harvested by centrifugation. Total RNA was isolated using the RNeasy Protect Bacteria Mini Kit (Qiagen, 74104) according to the manufacturer’s instructions. Plant nodule RNA was isolated from plant stem-nodules after inoculation with bacteria, at 3, 5, 7, and 10 dpi. Nodules were homogenized in liquid nitrogen with steel beads, and RNA was extracted with TRIzol Reagent. Total RNA from bacteroids was isolated from stem-nodules as follows. Stem-nodules were homogenized in liquid nitrogen with a mortar and pestle, and the paste was diluted with 1 ml ice-cold Mg-phosphate buffer and passed through a 40 μm filter (BD Biosciences) to collect the filtrate. The filtrate was centrifuged at 5,000 × *g* for 5 min at 4°C, and total RNA was extracted using TRIzol Reagent (Invitrogen, 15596018) according to the manufacturer’s instructions. To collect pure bacterial RNA, total RNA was purified using the MICROBEnrich Kit (Invitrogen, AM1901) according to the manufacturer’s instructions to obtain bacterial RNA. All of the above RNA samples were treated with TURBO DNase (Invitrogen, AM2238) to remove DNA contamination.

### Quantitative RT-PCR

Total RNA was isolated from free-living bacteria, bacteroids or plants as described above. First-strand cDNA was synthesized from the extracted RNA (1 μg) by reverse transcription (RT) using the SuperScript III First-Strand Synthesis System (Invitrogen) with the random primers (for bacteria) or oligo12-18 (for plants) included in the SuperScript III First-Strand Synthesis System according to the manufacturer’s instructions. The qRT-PCR was carried out with the LightCycler 480 system (Roche, Germany) and SYBR Green mix (KAPA Biosystems, United States). All primer sequences are listed in Table 2. The 16S rRNA gene was used as a reference gene for bacteria, and a ubiquitin cDNA fragment (*Srubi*) was used as a reference gene for the host plant to calculate the relative expression of each target gene. The data were processed with LightCycler 480 software (Version 1.5). All tests were performed at least in three independent biological replicates.

## Results

### ParA Protein Levels in *A. caulinodans* ORS571 Derivatives Cultivated in Either Rich (TY) or Minimal (L2-N) Medium

**FIGURE 1:**
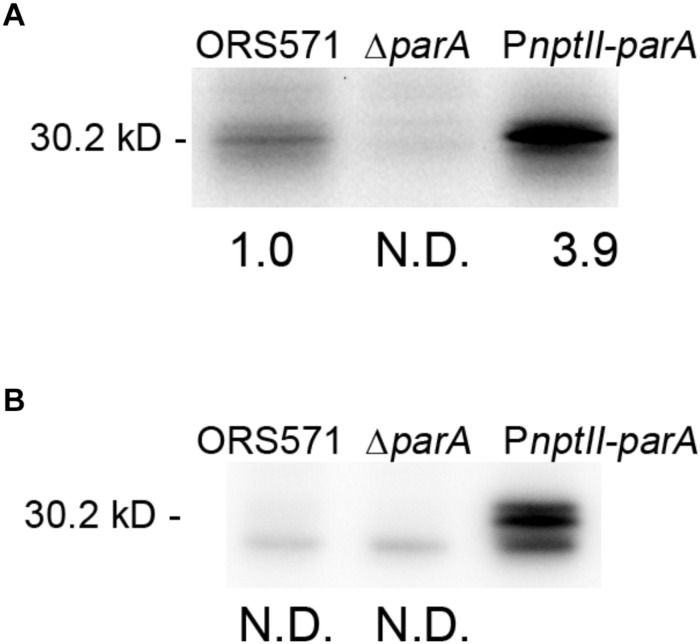
Quantitative Western blotting analysis. The relative protein quantity of ParA in *A. caulinodans* ORS571 (WT) and its derivatives. Soluble proteins were isolated from the ORS571, Δ*parA*, and P*nptII*-*parA* strains at the exponential phase (OD_600_ = 0.5) in TY medium **(A)** and at 15 h in L2 medium in the nitrogen-fixing state **(B)**. For quantification of endogenous ParA, the relative protein levels contents were calculated by measuring the intensity of each band with ImageJ. To ensure that the bands were in a linear range for densitometry, protein sample dilutions were prepared. N.D., not detected.

We constructed a ParA-overproducing *A. caulinodans* ORS571 strain (P*nptII-parA*) harboring a plasmid that constitutively expressed the *parA* gene under the control of the *nptII* promoter (Supplementary Figure S1). Western blot analysis was performed to examine the endogenous ParA protein level in the *parA* mutants cultivated in either TY or L2-N broth. When the bacteria were cultivated aerobically in rich broth (TY), the ParA protein level in P*nptII-parA* cells was significantly elevated (3.9-fold) compared with that in the wild-type strain (ORS571) (Figure 1A). In contrast, ParA was undetectable in the Δ*parA* cells. When the bacteria were cultivated in minimal broth (L2-N) under microaerobic conditions, high constitutive expression of the ParA protein was detected in the P*nptII*-*parA* cells (Figure 1B). However, ParA was not detected in the ORS571 cells, indicating that the level of protein content was below the detection limits.

### Overproduction of ParA Did Not Affect Growth, Cell Morphology, Chromosome Partitioning, or Vegetative Nitrogen Fixation in the Free-Living State

**FIGURE 2:**
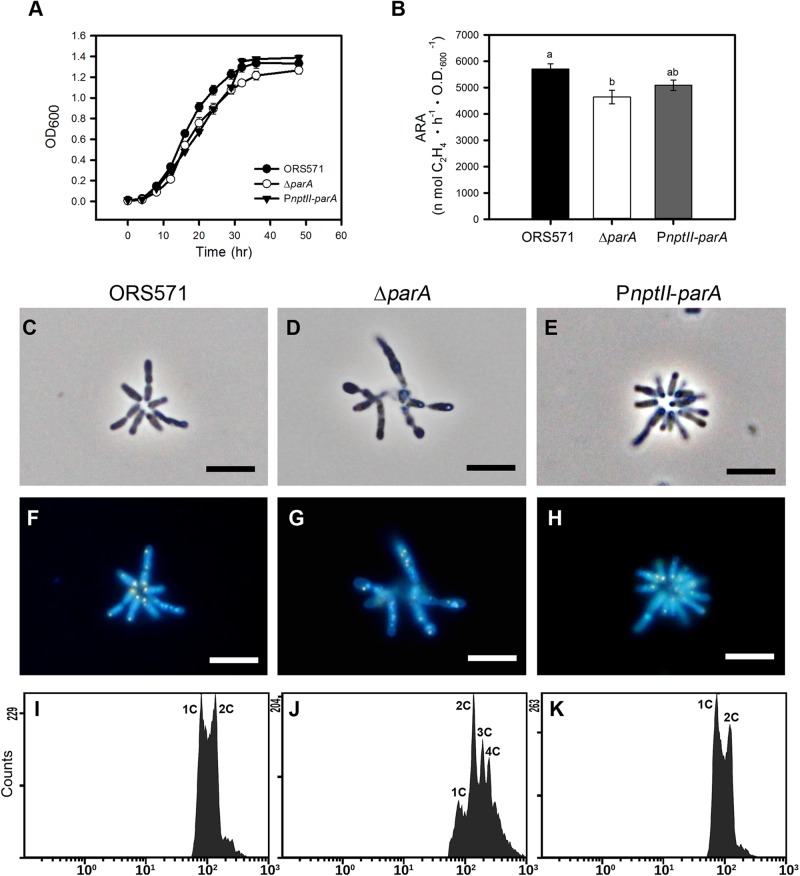
Phenotypes of *A. caulinodans* and its derivatives in the free-living state. **(A)** Growth curves of *A. caulinodans* and its derivatives under free-living conditions. All the bacteria were grown at 37°C in TY broth. The values are the means of at least three replicates. **(B)** Nitrogen fixation activity of *A. caulinodans* and its derivatives under free-living conditions. All the bacteria were grown for 15 h at 37°C in synthetic nitrogen-deficient medium (L2-N medium) to induce nitrogen fixation. The values are the means ± standard deviations of three biological replicates. *P* < 0.05 (*post hoc* analysis: Tukey). **(C–H)** Morphology of *A. caulinodans* derivatives under vegetative conditions. The ORS571 **(C,F)**, Δ*parA* **(D,G)**, and P*nptII*-*parA* **(E,H)** strains were incubated to OD_600_ = 0.5 in TY broth and stained with DAPI (10 μg/ml). **(C–E)** Phase contrast image. **(F–H)** Fluorescent image. Scale bars = 5 μm. **(I–K)** Flow cytometry analyses showing the DNA levels contents of the cultured bacteria. Exponential-phase cultures of ORS571 **(I)**, Δ*parA* **(J)**, and P*nptII*-*parA* **(K)** cells were fixed and stained with propidium iodide (PI). For each histogram, the *x* axis shows fluorescence levels, which represent the DNA levels per particle counted. The *y* axis shows counts, which indicate the number of fluorescing particles or cells. In each experiment, 20,000 cells were analyzed.

As shown in Figure 2A, the growth of P*nptII-parA* was slightly slower than that of ORS571 during the exponential phase when the bacteria were cultivated in TY broth, although the CFU did not differ from that of ORS571 (data not shown). The bacterial cells of P*nptII-parA* showed autoagglutination (rosetting), and the average length of each cell was 1.0 to 2.0 μm (Figure 2E). These morphological features were indistinguishable from those of ORS571 (Figure 2C). On the other hand, the growth rate of the *parA* gene null mutant (Δ*parA*) was reduced, which is consistent with the previous study (Liu et al., 2011). The Δ*parA* culture displayed large numbers of various sizes of filamentous and branched cells, and no autoagglutination was observed (Figure 2D). As shown in Figures 2F–H, the vegetative cells of both ORS571 and P*nptII*-*parA* contained compact nucleoids at the cell poles when stained with DAPI (4,6-diamidino-2-phenylindole). In contrast, the free-living Δ*parA* mutant cells were polyploid, suggesting dramatic defects in nucleoid partitioning (Figure 2G). We further performed flow cytometry analysis to confirm the DNA contents of the free-living cells in the exponential phase. As shown in Figure 2K, the DNA content distribution of P*nptII*-*parA* was composed of two peaks (1C and 2C), as in ORS571. On the other hand, multiple genome equivalents were observed in the Δ*parA* mutant cells (Figure 2J). These results are consistent with the microscopic observations. The vegetative nitrogen-fixing activity of the bacteria was determined by acetylene reduction assay (ARA), and the numeric value of the ARA was found to be comparable between P*nptII-parA* and ORS571 (Figure 2B), indicating that vegetative nitrogen-fixing ability is not affected by *parA* overexpression. In contrast, the ARA was significantly reduced in the Δ*parA* cells.

### Transcription of Selected Symbiotic Genes in the Free-Living State

To verify the transcription of symbiosis-related genes in the ORS571 derivatives in the vegetative state, we determined the expression levels of selected symbiosis-related genes, including nodulation genes (*nod*, *noe*, and *nol*), nitrogen-fixation genes (*nif*/*fix*), cell-cycle-related genes (*parA*, *dnaA*, and *ftsZ*), surface polysaccharide-related genes (*expA4*, *expA9*/*oac1* and *expA10*/*oac2*) and bacteroid differentiation related gene (*bacA*) in the ORS571 derivatives in the vegetative (i.e., grown in TY-rich medium under aerobic conditions) or free-living nitrogen-fixing state (i.e., grown in L2 minimal medium under microaerobic conditions). As shown in Table 3, the relative expression levels of the *parA* gene in P*nptII-parA* were considerably larger (83-fold) than those of the ORS571 in the nitrogen-fixing state. Strikingly, we noticed that the relative transcript levels of most of the target genes, especially those associated with nodulation (such as *nodD* and *nolK*), were elevated in P*nptII-parA* cells in the nitrogen-fixing state without flavonoid addition (Table 3). It has been proven that the flavonoid naringenin could induce the expression of nodulation genes in free-living *A. caulinodans* cells (Tsukada et al., 2009). For verification, we added the flavonoid naringenin to the broth (L2 + N medium) and determined the transcription levels of some nodulation-related genes (*nod* and *noe*) (detailed in Supplementary Materials). As shown in Supplementary Figure S2, the addition of naringenin significantly elevated the expression of the genes in ORS571. This result was consistent with the previous finding reported by Tsukada et al. (2009). On the other hand, we found only the expression of the *nodD* gene was enhanced in the vegetative P*nptII-parA* cells (Supplementary Figure S2). In contrast, the expression of those of the other nodulation related genes were declined. Similarly, these genes were all repressed in the Δ*parA* cells.

**TABLE 3:** Bacterial gene expression in the free-living state.

		**TY medium**		**L2-N medium**	
			
**Category**	**Gene name**	**Δ*parA*/WT**	**P*nptII-parA*/WT**	**Δ*parA*/WT**	**P*nptII-parA*/WT**
nod	*nodD*	5.1 ± 0.62^∗^	1.02 ± 0.23	1.37 ± 0.27	5.48 ± 0.17^∗^
	*noeC*	6.87 ± 1.32^∗^	0.52 ± 0.38	1.78 ± 0.47^∗^	1.84 ± 0.63
	*nodZ*	16.29 ± 7.20^∗^	0.49 ± 0.34	0.60 ± 0.11^∗^	2.38 ± 0.77^∗^
	*nodB*	11.44 ± 1.08	0.69 ± 0.46	0.58 ± 0.11^∗^	3.43 ± 0.73^∗^
	*nolK*	6.58 ± 0.34^∗^	0.81 ± 0.77	2.51 ± 0.75	2.19 ± 0.15^∗^
nif/fix	*nifD*	1.35 ± 0.44	0.43 ± 0.15^∗^	0.89 ± 0.24^∗^	1.89 ± 0.18^∗^
	*nifH2*	1.95 ± 0.03	0.42 ± 0.26	0.71 ± 0.13^∗^	2.47 ± 0.06^∗^
	*nifA*	1.96 ± 0.46	0.96 ± 1.14	0.85 ± 0.19^∗^	0.03 ± 0.02
	*fixN*	0.57 ± 0.50	0.25 ± 0.09^∗^	1.40 ± 0.23^∗^	1.33 ± 0.06^∗^
Polysaccharide	*oac3/expA7*	0.87 ± 0.21	0.44 ± 0.14^∗^	0.55 ± 0.11	2.68 ± 1.13
	*oac2/expA10*	0.98 ± 0.22	0.83 ± 0.3	0.44 ± 0.12^∗^	2.78 ± 0.56^∗^
	*oac1/expA9*	0.95 ± 0.36	1.28 ± 0.44	0.88 ± 0.18	2.50 ± 0.16^∗^
	*expA4*	2.61 ± 2.53	0.36 ± 0.26	0.67 ± 0.10^∗^	3.01 ± 0.16^∗^
Cell-cycle-related genes	*ftsZ*	1.74 ± 0.01^∗^	1.09 ± 0.48	0.67 ± 0.14	2.66 ± 0.09^∗^
	*parA*	1.43 ± 0.03	28.66 ± 14.17^∗^	1.76 ± 0.09^∗^	83.74 ± 3.38^∗^
	*parB*	1.75 ± 1.36	1.07 ± 0.56	0.51 ± 0.04^∗^	1.87 ± 0.15^∗^
	*dnaA*	3.81 ± 2.79	3.98 ± 1.41^∗^	3.61 ± 3.27	0.71 ± 0.53
Bacteroid differentiation related gene	*bacA*	1.63 ± 1.35	1.61 ± 0.64	3.41 ± 0.27^∗^	2.00 ± 0.33^∗^

*^∗^*T*-test *P* < 0.05.*

### *parA* Overexpression Caused Aberrant Stem-Nodules

**FIGURE 3:**
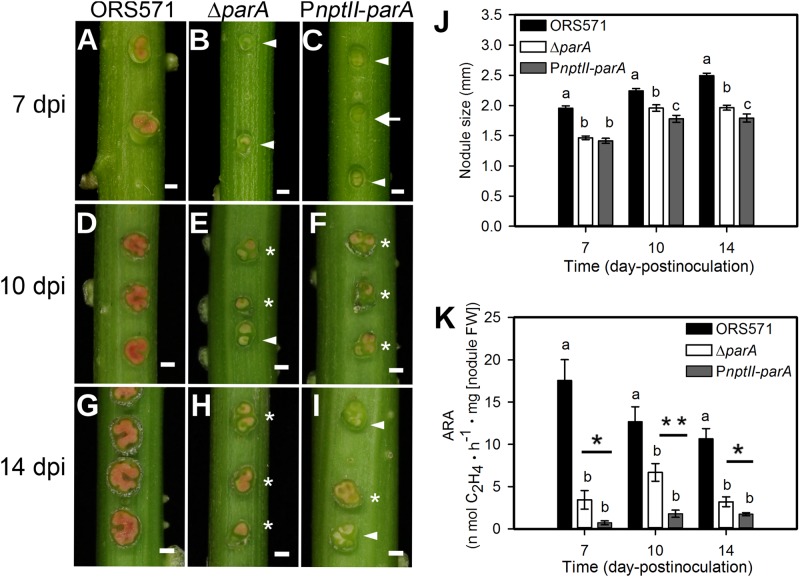
Phenotypes of *S. rostrata* stem nodules induced by *A. caulinodans* and derivatives. **(A–I)** Stem nodules induced by ORS571 **(A,D,G)**, Δ*parA* **(B,E,H)**, and P*nptII*-*parA* **(C,F,I)**. Arrowheads indicate beige stem nodules, asterisks mark pale red stem nodules, and arrows indicate green stem nodules. The scale bar represents 1 mm. **(J)** The sizes of the stem nodules formed by ORS571 (black), Δ*parA* (white) and P*nptII-parA* (gray) were calculated as the means of 30 nodules. The values are the means ± standard deviations of five biological replicates. *P* < 0.05 (*post hoc* analysis: Tukey). **(K)** Nitrogen-fixing activities of stem nodules. The stem nodules formed by each strain were harvested at 7, 10, and 14 dpi and their nitrogenase activities were measured by the acetylene reducing assay (ARA). The values are the means ± standard deviations of five biological replicates. *P* < 0.05 (*post hoc* analysis: Tukey’s HSD test). To distinguish the difference between the two mutants, a Student’s *t*-test was further performed. ^∗^*P* < 0.05, ^∗∗^*P* < 0.05.

We inoculated the ORS571 derivatives onto the stems of *S. rostrata*, and all plants were grown at 35∼40°C under a 24-h light regime. As shown in Figure 3J, the average size of the 14 dpi P*nptII-parA* stem nodules was approximately 1.8 mm, significantly smaller than that of ORS571 (2.49 mm). The cross-sections of the 7 dpi P*nptII-parA* stem nodules showed green or beige interiors (Figure 3C), whereas those of ORS571 were pink or red (Figure 3A). As shown in Figures 3F,I, some of the P*nptII-parA* stem-nodules appeared pale red (asterisks) or beige (arrowheads). We determined the nitrogen-fixing activity (ARA) of the stem-nodules and found that the P*nptII-parA* stem-nodules showed greatly reduced ARA during the entire nodulation period (Figure 3K).

**FIGURE 4:**
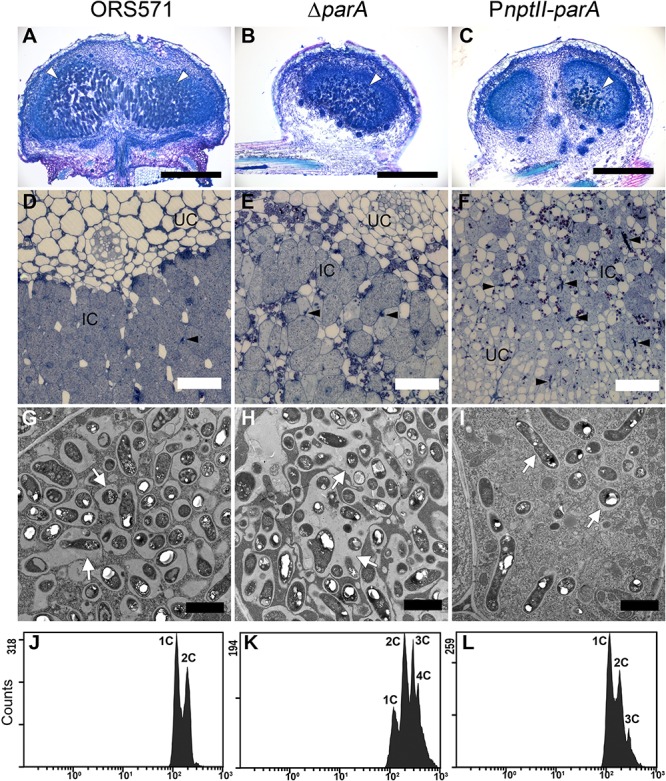
Cytological characterization of 7-dpi stem nodules and their DNA contents in bacteroids. Stem nodules (7 dpi) formed by ORS571, Δ*parA*, and P*nptII-parA*. **(A–C)** Light micrographs of longitudinal sections stained with toluidine blue O. White arrowheads indicate infection centers. **(D–F)** High magnification light micrographs. Black arrowheads indicate infection threads. **(G–I)** Transmission electron microscope (TEM) images. Arrows indicate peribacteroid membrane spaces. IC, infected cells; UC, uninfected cells. Scale bars represent 0.5 mm in panels **(A–C)**, 50 μm in panels **(D–F)**, and 2 μm in panels **(G–I)**. **(J–L)** Bacteroids were isolated from 7 dpi stem nodules and stained with propidium iodide (PI) for flow cytometry analyses. For each histogram, the *x* axis shows fluorescence levels, which represent the DNA content per particle counted. The *y* axis shows counts, which indicate the number of fluorescing particles or cells. In each experiment, 20,000 cells were analyzed.

In the stem-nodules of ORS571 or Δ*parA*, large numbers of bacteria colonized the infection centers (i.e., the dark blue regions stained by toluidine blue O) (Figures 4A,B,D,E). In contrast, fewer and smaller infected cells were present in the central cortical tissues of the P*nptII-parA* stem-nodules (Figures 4C,F). In addition, some infection threads proliferated abnormally, forming reticulated architectures, and only a few internalized bacteria were observed in the cortical cells (Supplementary Figure S3).

In the P*nptII-parA* mutant-induced nodules, the symbiosome membrane closely surrounded a single pleomorphic-shaped bacteroid (Figure 4I), whereas a broad symbiosome space between the bacteroid(s) and the symbiosome membrane was observed in both the ORS571 (Figure 4G) and Δ*parA* infected nodules (Figure 4H). We also performed flow cytometry analysis to determine the DNA contents of the bacteroids in the different stem-nodules. In the 7 dpi stem nodules induced by the two *parA* mutants (Δ*parA* and P*nptII-parA*), triploid or tetraploid (3C or 4C) cells were able to be observed in the bacteroids (Figures 4K,L). On the other hand, ORS571 bacteroids were mainly haploid and diploid (1C and 2C) (Figure 4J).

### Expression of Symbiosis-Related Genes in Bacteroids

To verify the symbiotic gene expression of the ORS571 derivatives in the early stages of nodulation, we detected the transcripts of certain bacterial genes related to the early stage of stem-nodule development by qPCR. As shown in Table 4, the relative expression of these cell-cycle-related genes, such as *parA* and *ftsZ*, was upregulated in both Δ*parA* and P*nptII*-*parA* nodules, especially that of the *parA* gene, which was dramatically elevated in the latter. Notably, the level of *dnaA*, a key bacterial DNA replication initiation factor, was significantly increased in only the P*nptII*-*parA* nodules. We also determined the transcript levels of nodulation related genes (*nod*, *noe* and *nol*). In general, when the regulatory gene *nodD* is activated, it leads to the expression of the other nodulation related genes (Mergaert et al., 1996). We found that the *nod*D gene was downregulated in the P*nptII*-*parA* nodules; however, some other nodulation genes, such as *nod*B, *nod*Z, and *noe*C, were upregulated. This result indicates that the expression of these genes was uncoupled from normal regulation. Moreover, this phenomenon could be corresponding to the finding shown in Supplementary Figure S2.

**TABLE 4:** Gene expression in 5 dpi bacteroids.

		**Gene expression**	
		
**Category**	**Gene name**	**Δ*parA*/WT**	**P*nptII-parA*/WT**
nod	*nodD*	0.93 ± 0.03	0.37 ± 0.10^∗^
	*noeC*	5.28 ± 0.10^∗^	1.36 ± 0.20
	*nodZ*	5.96 ± 0.27^∗^	1.59 ± 0.23^∗^
	*nodB*	9.29 ± 1.55^∗^	5.67 ± 0.88^∗^
	*nolK*	4.18 ± 0.06^∗^	0.53 ± 0.01^∗^
nif/fix	*nifD*	2.68 ± 0.18^∗^	0.63 ± 0.03^∗^
	*nifH2*	1.39 ± 0.01^∗^	0.03 ± 0.00^∗^
	*nifA*	1.09 ± 0.07	0.24 ± 0.25^∗^
	*fixA*	0.26 ± 0.01^∗^	0.04 ± 0.00^∗^
	*fixN*	0.59 ± 0.06^∗^	0.50 ± 0.08^∗^
Polysaccharide	*oac3/expA7*	7.57 ± 5.22	1.74 ± 1.22
	*oac2/expA10*	2.27 ± 0.15^∗^	1.28 ± 0.04^∗^
	*oac1/expA9*	1.67 ± 0.36	0.53 ± 0.03^∗^
	*expA4*	6.64 ± 0.32^∗^	5.00 ± 0.38^∗^
Cell-cycle-related genes	*ftsZ*	1.55 ± 0.08^∗^	1.73 ± 0.03^∗^
	*parA*	2.05 ± 0.25^∗^	21.06 ± 0.18^∗^
	*parB*	1.79 ± 0.43^∗^	0.68 ± 0.07^∗^
	*dnaA*	1.01 ± 0.46	3.18 ± 0.12^∗^
Bacteroid differentiation related gene	*bacA*	1.58 ± 0.04^∗^	1.69 ± 0.21^∗^

*^∗^*T*-test *P* < 0.05.*

Based on the observed transcript levels, we deduced that the aberrant phenotype of the two *parA* mutant-induced nodule-like structures was due to a lack of coordination with the developmental stages of the host plant. We also noted that the transcripts of the symbiotic nitrogen-fixation genes *nif* and *fix* were repressed in the P*nptII*-*parA* stem nodules. However, only *fix* gene transcripts were reduced in the Δ*parA* stem-nodules. These findings may explain the dramatic decrease in symbiotic nitrogen fixation activity in the plants elicited by the two *parA* mutants and the result that the decrease associated with P*nptII*-*parA* was more severe than that of Δ*parA* (Figure 3K). In rhizobia, surface polysaccharides (LPS, EPS, SPS, etc.) are also important for the establishment of effective symbiotic interactions with host legumes. We noticed that the expression levels of the EPS-production-related gene (*expA4*) in the Δ*parA* and P*nptII*-*parA* nodules were approximately 5- to 6-fold higher than those in the ORS571 nodules (Table 4). It has been proved that adequate amount of azorhizobial EPSs is indispensable for *S. rostrata* stem-nodule development (Gao et al., 2001; Mathis et al., 2005; Sato et al., 2016). Accordingly, we deduced that the increased amount of EPSs is one of the factors resulted in aberrant phenotypes of the *parA* mutants. How the azorhizobial ParA protein involves in the regulation of exopolysaccharide production remains to be elucidated.

### RT-PCR Analyses of Plant Defense Response-Related Genes

**FIGURE 5:**
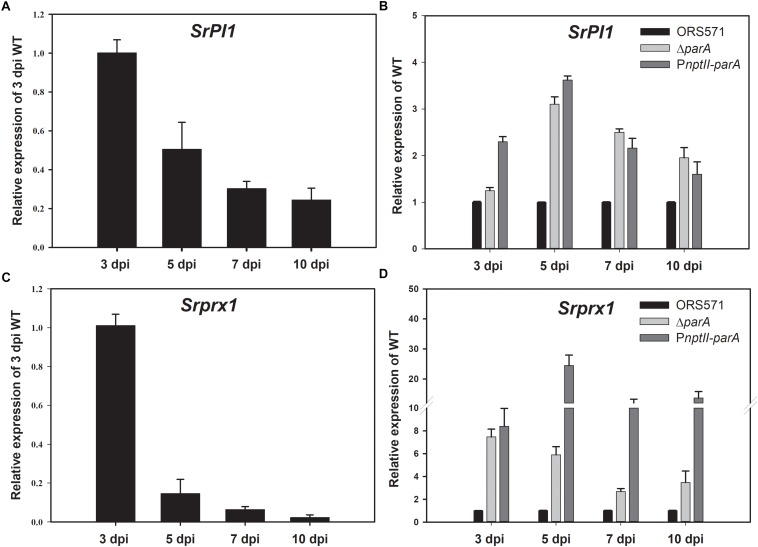
Plant defense response-related genes. Expression of the plant immune response-related genes *SrPI1* and *Srprx1* during nodulation. *SrPI1* expression pattern in ORS571-inoculated plants is shown as relative mRNA expression compared to 3 dpi ORS571 **(A)**, and *SrPI1* expression in *ΔparA* and P*nptII-parA* is shown as relative expression compared to ORS571 at the same time point **(B)**. **(C)** *Srprx1* expression profile of ORS571 shown as relative mRNA expression compared to 3 dpi ORS571. **(D)** *ΔparA* and P*nptII-parA Srprx1* gene expression shown as relative mRNA expression compared to ORS571 at the same time point. The values are the means ± standard deviations of three biological replicates.

During the early stage of stem nodulation in *S. rostrata*, a Kunitz proteinase inhibitor (*SrPI1*) gene is expressed as a part of the plant defense mechanism (Lievens et al., 2004). As shown in Figure 5A, the transcript levels of *SrPI1* were upregulated and reached a maximum at the early stage of nodulation (3 dpi) and then decreased during nodule development, which is consistent with previous studies (Lievens et al., 2004). When the strains were inoculated on the stem of *S. rostrata*, the relative transcript levels of the *SrPI1* gene in both the Δ*parA* and P*nptII*-*parA* nodules were higher than those of the wild type (Figure 5B) during nodule development (3–10 dpi), especially those of P*nptII*-*parA*.

*Srprx1*, which encodes a functional class III peroxidase isoform of *S. rostrata*, is also transiently expressed in the early stage of stem nodulation (Den Herder et al., 2007). Its expression is induced by compatible nodulation factors (NFs) and is affected by H_2_O_2_ produced in the bacterial infection pockets and infection threads during infection. As shown in Figure 5C, the transcript level of *Srprx1* decreased during nodule development. However, the relative transcript levels of the *Srprx1* gene induced by Δ*parA* or P*nptII*-*parA* were all dramatically higher than those elicited by the ORS571 during nodule development (3–10 dpi) (Figure 5D). This result suggests that the intracellular H_2_O_2_ concentrations during nodulation in Δ*parA* and P*nptII-parA* stem-nodules (3–10 dpi) are higher than those in the wild-type strain.

## Discussion

Chromosomal Par proteins have been studied in many bacteria, and their absence or overproduction causes defects in segregation or/and cellular processes that include chromosome replication and cell division (Draper and Gober, 2002; Schofield et al., 2010). In our previous study, we found that a *parA* gene (AZC_4711) in-frame deletion mutant of *A. caulinodans* ORS571 (Δ*parA*) showed a pleiomorphic cell shape phenotype and was polyploid, with differences in nucleoid sizes (Liu et al., 2011). In this study, overexpression of the *parA* gene in ORS571 resulted in minor effects in the free-living state (Figure 2). On the other hand, it caused severe aberrant symbiotic phenotypes, indicating this mutation exerts specific effects on symbiosis.

As shown in Figure 2, the viability, morphology and DNA content of this bacterium (P*nptII-parA*) were indistinguishable from those of ORS571 cells. The P*nptII-parA* strain harbored a plasmid that expressed the *parA* gene constitutively under the control of the *nptII* promoter, and the ParA protein level in this bacterium was indeed significantly elevated in comparison with that of the wild-type strain (ORS571) (Figure 1). It has been considered that bacterial chromosome partitioning and cell division are tightly connected cellular processes. To maintain bacterial shape, cytoskeletal element genes, such as *ftsZ* and *mreB*, should be tightly regulated during cell division (Cabeen and Jacobs-Wagner, 2005). Generally, overproduction of chromosome partitioning proteins in most bacteria leads to changes in morphology and the cell cycle, with varying severity. For example, increasing ParA proteins in *C. crescentus* resulted in elongated cells and cell division defects (Mohl and Gober, 1997). In *P. aeruginosa*, overexpressing the native *parA* gene at different levels led to morphological changes (filamentous or enlarged cells) and strong growth defects (Lasocki et al., 2007). In addition, the population of anucleate cells was increased, and the cell motility was dramatically affected (Lasocki et al., 2007). In *B. subtilis*, overproduction of the ParA ortholog (Soj) caused aberrant replication initiation, resulting in elongated cells harboring expanded nucleoids (Ogura et al., 2003). However, overexpression of a ParA ortholog (SegA) in an archaeon, *Sulfolobus solfataricus*, did not affect the normal growth rate, although severe chromosome segregation defects occurred (Kalliomaa-Sanford et al., 2012). Genetic robustness or genetic compensation is a phenomenon conserved in numerous prokaryotes and eukaryotes, which require buffering systems to ensure similar developmental outcomes despite minor differences in genetic makeup or environmental conditions (El-Brolosy and Stainier, 2017). The loss or overexpression of related gene(s), especially for those involved in tightly regulated cellular networks, may be compensated by others with overlapping functions (i.e., functionally redundant genetic pathways) and expression patterns (i.e., transcriptional modulation/transcriptional adaptation) to overcome stochastic fluctuations in gene and protein expression (Tautz, 1992; El-Brolosy et al., 2018). Accordingly, we deduced that one or more paralogous genes or underlying mechanisms provide functional physiological redundancy to maintain the morphology of P*nptII-parA* cells.

Notably, when P*nptII-parA* was cultivated in minimal broth (L2-N) under microaerobic condition (i.e., nitrogen-fixing state), this strain revealed a very high expression level of the *parA* gene (Table 3). In general, the expression levels of nodulation genes in vegetative azorhizobial cells are induced only when a flavonoid (naringenin) is added to the culture medium (Tsukada et al., 2009). However, we noticed the expression levels of some symbiosis-related genes, including nodulation genes, nitrogen-fixation genes, and surface polysaccharide-related genes, were drastically induced in the nitrogen-fixing state without flavonoid addition (Table 3). In contrast, when P*nptII-parA* was cultivated with naringenin, only *nodD* gene was enhanced, those of the other target nodulation genes were declined (Supplementary Figure S2). It suggests that the nodulation genes in the P*nptII-parA* mutant were not mediated normally by the plant flavonoid. This phenomenon was reminiscent of the spontaneous flavonoid independent transcription activation (FITA) mutants of *nodD* in *Sinorhizobium meliloti* (Spaink et al., 1989) and in *S. fredii* (Vinardell et al., 2004). Legume roots secrete flavonoids, which are specifically recognized by NodD and bind to *nod* boxes and activate the transcription of *nod* genes (Maj et al., 2010). Expression of *nod* genes results in the production and secretion of Nod factors (lipochitooligosaccharides) and then initiate the nodulation program. *A. caulinodans* ORS571 *nod* genes are known to be induced by specific *S. rostrata* flavonoids (Goethals et al., 1990). Taken together, we deduced that the P*nptII-parA* cells initiated symbiosis in *S. rostrata* via a still-unidentified flavonoid-independent pathway, causing a nodule-like structure and impaired symbiotic nitrogen fixation (Figure 3). Further work to elucidate the underlying mechanism of this phenomenon, such as constructing a gain-of-function mutant (i.e., Δ*nodD* harbored with *PnptII-parA* plasmid) to conduct genetic and physiological analyses remains to be done.

After rhizobia are released from the infection threads (ITs), they reside in the host cytoplasm as organelle-like structures, called symbiosomes. They comprise the bacteroids, the peribacteroid membrane (symbiosome membrane) and the space between them (Defaria et al., 1986). As shown in Figure 4I, we observed that individual bacterium was closely surrounded by the peribacteroid membrane in one symbiosome, distributed in the cytoplasm of P*nptII-parA* nodules. The nitrogen-fixing ability (C_2_H_2_-reduction) of the stem-nodules was confirmed by ARA measurement, however, the values were greatly reduced compared with the wild type (Figure 3K). It was also consistent with the transcription of nitrogen fixation genes (*nif* and *fix*) in the P*nptII*-*parA* stem nodules (Table 4). According to microscopy, there were several enlarged ITs and only a few internalized bacteria observed in the cortical cells (Figure 4 and Supplementary Figure S3). It has been known that rhizobia don’t fix nitrogen within ITs until they differentiate into bacteroids in symbiosomes. Taken together, we deduced that a majority of P*nptII-parA* was retained within the ITs in the analyzed nodules, and only a small portion of this bacterium could successfully release from the ITs and differentiate into nitrogen-fixing bacteroids.

As shown in Figure 4G, one or multiple ORS571 bacteroids with a broad symbiosome space were enclosed within the symbiosome compartment in the 7 dpi-old-stem-nodules. These wild type bacteroids were mainly haploid and diploid (1C and 2C) (Figure 4J). In the fully mature stage (14 dpi), they would become polyploid (2C, 3C, and 4C), but the space between the symbiosome membrane and bacteroids were still large (Liu et al., 2011). In contrast, the P*nptII-parA* symbiosome had a remarkable trait, in which each single pleomorphic-shaped bacteroid was closely surrounded by a peribacteroid membrane in the P*nptII-parA* stem-nodules (7 dpi) (Figure 4I). Besides, elevated DNA content was already observed in the P*nptII-parA* bacteroids, although it was not as marked as that in the Δ*parA* bacteroids (Figure 4L). We noticed these distinctive features of P*nptII-parA* bacteroid (i.e., single swollen bacteroid, relatively narrow symbiosome space, and multinucleoid [polyploid] cells, *etc*.) are reminiscent of the terminally differentiated bacteroids in some IRLC indeterminate nodules (Mergaert et al., 2006; Jones et al., 2007; Oono et al., 2010). To the best of our knowledge, there have been no previous reports of such bacteroid morphotypes within the root or stem nodules of *S. rostrata*.

Many studies have shown that bacteroid differentiation fates (terminal differentiation) are mediated by plant factors that show similarities to defensin-like innate immunity factors and have been designated as NCR peptides; these factors are present in the nodules of IRLC and Dalbergoid (*Aeschynomene* spp.) clade legumes (Van De Velde et al., 2010; Czernic et al., 2015). *S. rostrata* has been classified as a member of the Robinioid clade in the Papilionoideae (Sprent, 2007), implying that its nodules do not produce NCR peptides. The mature stem-nodules of *S. rostrata* are regarded as determinate (Goormachtig et al., 1997), meaning that the endosymbionts will divide after a time within the plant membrane and form one or multiple bacteroids with a broad symbiosome space via peribacteroid membrane fusion or further bacterial division (Brewin, 2004). Such symbiosomes are also observed in *Lotus japonicus*, soybean (*Glycine max*), bean (*Phaseolus vulgaris*), etc (Kereszt et al., 2011). Van de Velde and colleagues reported that expression of heterologous NCR genes (derived from IRLC legume) in *L. japonicus* would also generate polyploid and enlarged *Mesorhizobium loti* bacteroids with a narrow symbiosome space within the majority of symbiotic cells (Van De Velde et al., 2010). Terminal bacteroid differentiation is a cell-cycle-related process, and the formation of the single swollen bacteroids present in symbiosomes is known to be mediated by NCR peptides that affect the bacterial cell cycle, provoke membrane modifications, inhibit bacterial cytokinesis, and promote DNA amplification coupled with cell enlargement (Mergaert, 2018). In many bacteria, deletion or overproduction of the chromosomal *par* genes results in some defects in segregation and/or cellular processes (Draper and Gober, 2002; Schofield et al., 2010). As shown in Table 4, the relative transcript level of the DNA replication*-*related gene *dnaA* was significantly upregulated in the P*nptII-parA* bacteroids, and this phenomenon may be related to their polyploidy (Figure 4L). Because *S. rostrata* is not supposed to produce NCR peptides, we propose that the formation of this symbiosome type in the P*nptII-parA* stem-nodules is due to the severe cell cycle disturbance elicited by the excess of ParA proteins in the symbiotic cells during nodulation.

As shown in Supplementary Figure S3, several abnormally proliferated infection threads were formed in the central cortical tissues of the P*nptII-parA* stem-nodules. A gibberellin (GA) biosynthesis related gene, *SrGA20ox1*, was known to be involved in infection thread formation in *S. rostrate* (Lievens et al., 2005). Its transcript was up-regulated during early nodulation of stem-nodule. Accordingly, we conducted RT-PCR analysis to determine the *SrGA20ox1* expression. As shown in Supplementary Figure S4A, the transcript levels of ORS571 infected nodule were upregulated and reached a maximum at the early stage of nodulation (3 dpi) and then decreased during nodule development, and almost disappeared in the 7 dpi stem-nodule. It indicates that the formation of infection threads was active at early stage, and stopped in subsequent stages of nodule development. On the other hand, the relative transcripts of P*nptII*-*parA* infected nodule kept at relatively high levels during nodule development (3–7 dpi) (Supplementary Figure S4B). It suggests that the huge number of large infection threads in the P*nptII*-*parA* nodule was due to persistent induction of infection threads expansion and invagination. In general, signs of defense could be associated with an attempt by the plant to limit bacterial multiplication. The enlarged infection threads also suggest the induction of a plant defense response accompanied with massive H_2_O_2_ accumulation (D’haeze et al., 2004). As shown in Figure 5, the relative transcript levels of the plant defense response-related genes (*SrPI1* and *Srprx1*) induced by P*nptII*-*parA* were dramatically higher than those elicited by the ORS571 during nodule development (3–10 dpi). It suggests that the stem-nodules induced by P*nptII-parA* mutant exhibited higher levels of oxidative stress than those induced by wild type.

In a preliminary experiment, we found that the EPS production of the ORS571 derivatives was remarkably increased at high concentration of H_2_O_2_ (5 mM) (upper panel of Supplementary Figure S5). We noticed that the viability of individual strains under the same dosage of hydrogen peroxide treatment was approximately the same (middle panel of Supplementary Figure S5). We further determined the outer membrane permeabilities of the ORS571 derivatives by propidium iodide (PI) staining. As shown in bottom panel of Supplementary Figure S5, we found the cell permeability of the P*nptII-parA* mutant was dramatically higher than that of either wild type or Δ*parA* mutant whether it is treated with H_2_O_2_ or not. In general, the increased membrane penetrability is the direct evidence of cell membrane damage in the response to stress. It indicates that P*nptII-parA* cells are supposed to be more easily damaged by the environmental stress than the other two strains. Unexpectedly, the tolerance of P*nptII-parA* toward H_2_O_2_ was comparable with ORS571 (middle panel of Supplementary Figure S5). It has been proposed that massive H_2_O_2_ accumulation was induced by P*nptII-parA* mutant during nodulation, while the expression level of the EPS-production-related gene (*expA4*) of the P*nptII*-*parA* bacteroids was significantly higher (∼5 folds) than that of the ORS571 bacteroids (Table 4). Since EPS synthesized by bacteria are thought to be adaptations to environmental stresses (Lloret et al., 1998), it is likely that P*nptII-parA* cells may mitigate the oxidative stress by elevated levels of EPS production during nodulation.

Some features of P*nptII*-*parA* nodule phenotype were reminiscent of ORS571-oac2 mutant induced stem-nodule (Gao et al., 2001; Mathis et al., 2005). Strain ORS571-oac2 has truncated LPS, and produces less EPS in comparison with that of wild type. The development of ORS571-oac2 induced stem-nodule was arrested at early stage. There were several enlarged, thick-walled infection threads formed, which was due to the infection steps continuously repeated in the nodule. Notably, there were also some features different from those of P*nptII-parA* stem-nodules. For example, bacterial exit from infection threads was completely blocked in the ORS571-oac2 stem-nodule (Mathis et al., 2005), whereas bacterial internalization and bacteroid formation was observed in the central cortical tissue of P*nptII-parA* stem-nodules (Figure 4). Besides, no nitrogen-fixing activity was detected in the former (Gao et al., 2001), in contrast, nitrogenase enzyme activity (ARA) was detected in the latter although it was dramatically reduced (Figure 3K).

## Conclusion

Based on the phenotypic and gene expression analysis data in this study, we deduced that the particular symbiotic phenotype induced by P*nptII-parA* mutant is due to a lack of coordination with the developmental stages of the host plant. It results in polymorphic infection and organogenesis patterns, such as earlier occurring problem during the infection process and/or release from ITs as well as altered bacteroid differentiation. Since both the deletion and overexpression of the *A. caulinodans parA* gene result in aberrant symbiotic phenotypes and remarkably reduced performance, indicating ParA protein homeostasis should be tightly regulated in *A. caulinodans* at the correct level.

## Perspectives

It has been widely accepted that bacteroid morphology (swollen or non-swollen) is controlled by legume host factors rather than rhizobial genotype (Oono et al., 2010). In this study, we characterized different bacteroid formation traits (swollen and non-swollen) that were induced by two azorhizobial strains with the same genotype (ORS571 and *PnptII-parA*) in the same legume host (*S. rostrata*). This symbiotic system could serve as an excellent model for investigating the molecular mechanisms by which host legumes recognize and discriminate among effective and ineffective rhizobial strains. Furthermore, we could also explore the adaptation mechanisms of the same rhizobial strain within different types of symbiosomes, which will help to identify additional determinants of the interactions between host cells and bacteroids.

## Data Availability Statement

The datasets generated for this study are available on request to the corresponding author.

## Author Contributions

H-LC carried out most of the experiments, experimental data analysis, and manuscript writing. W-ZH and M-YT constructed the *parA* overexpression mutant. W-ZH analyzed the ParA protein expression level. C-HC provided technical assistance with TEM. C-TL is the corresponding authors in charge of the project design, and manuscript writing.

## Conflict of Interest

The authors declare that the research was conducted in the absence of any commercial or financial relationships that could be construed as a potential conflict of interest.
