# Supplementary material for: Overexpression of the Chromosome Partitioning Gene parA in Azorhizobium caulinodans ORS571 Alters the Bacteroid Morphotype in Sesbania rostrata Stem Nodules
Source: Front Microbiol. 2019 Oct 24;10:2422. doi: 10.3389/fmicb.2019.02422 (PMC6842974; doi:10.3389/fmicb.2019.02422)
Supplement: Supplementary file 2 [file Data_Sheet_1.pdf]

Overexpression of the Chromosome Partitioning Gene *parA* in *Azorhizobium caulinodans* ORS571 Alters the Bacteroid Morphotype in *Sesbania rostrata* Stem Nodules

Hsiao-Lin Chien[1], Wan-Zhen Huang[1], Ming-Yen Tsai[1], Chiung-Hsiang Cheng[2], Chi-Te Liu*[1,3]

[1]Institute of Biotechnology, National Taiwan University, Taipei, 106, Taiwan

[2]Institute of Molecular and Comparative Pathobiology, School of Veterinary Medicine, National Taiwan University, Taipei, 106, Taiwan

[3]Agricultural Biotechnology Research Center, Academia Sinica, Taipei, 115, Taiwan

Corresponding author:

*Chi-Te Liu

e-mail: chiteliu@ntu.edu.tw

Supplementary Information

Materials and methods

H_2O_2 sensitivity assay

Bacteria were grown at 37°C in the L2+N broth (i.e L2-N medium supplemented with $(NH_4)_2SO_4$, 0.04 g/liter), up to an optical density at 600 nm (OD_{600}) of 0.1. The flavonoid naringenin (20 μM) was added to the broth and incubated for 6 hours to mimic the induction of the nodulation process as described by (Tsukada et al., 2009). The broth was then treated with H_2O_2 to a final concentration of 1, 3, or 5 mM, respectively, and incubated at 37°C for thirty minutes in accordance with a previously described method (Lehman and Long, 2013). Bacterial cell viability was measured by determining the optical density (OD_{600}) or number of colony-forming units. The survival rate was determined as the percentage of survival of H_2O_2-treated versus untreated cultures.

Isolation and quantification of EPS

Exopolysaccharides (EPS) of ORS571 derivatives were determined using the phenol-sulfuric acid method (Dubois et al., 1956). Cells treated with H_2O_2 were removed from culture by centrifugation for 20 min at 12,000 ×g. The EPS was precipitated from the supernatant by the addition of 3 volumes of cold acetone. The pellet was resuspended in water, 5.0 % phenol and 96 % sulfuric acid at a ratio of 1:1:5 and mixed well. The solution was incubated for 20 min, and measured at a wavelength of 490 nm. The amount of EPS (mg) was calculated using a glucose standard calibration curve.

Bacterial membrane permeabilization assay

The outer-membrane permeability of the respective ORS571 derivative was measured in accordance with the method proposed by (Mergaert et al., 2006). Bacteria were grown at 37°C in the L2+N broth (i.e L2-N medium supplemented with $(NH_4)_2SO_4$, 0.04 g/liter), up to an optical density at 600 nm (OD_{600}) of 0.2. The broth was then treated with H_2O_2 to a final concentration of 0 or 5 mM, respectively, and incubated at 37°C for thirty minutes in accordance with a previously described method (Lehman and Long, 2013). Cells were collected by centrifugation for 2 min at 5,000 ×g and washed twice with PBS buffer. The pelleted cells were stained with propidium iodide (PI). The cells in suspension were mounted and examined by light microscopy under a bright or fluorescent field by a U-MWU2 filter set with UV excitation (excitation wavelength, 330 to 385 nm; emission wavelength, 420 nm; Olympus, Japan). In each experiment, over 1,000 cells were analyzed.

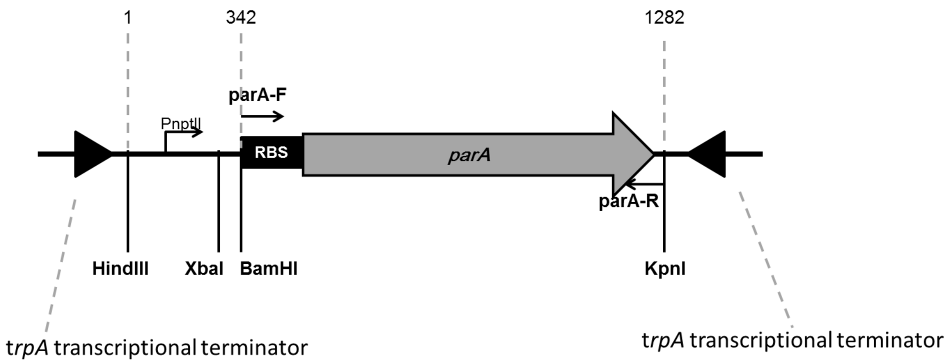

Figure S1. Gene map of the *parA* gene constitutive expression allele P*nptII-parA*.

The *parA* overexpression strain ORS571-P*nptII-parA* was constructed via ligation of the indicated PCR product into pFAJ1708, as described in the Materials and Methods.

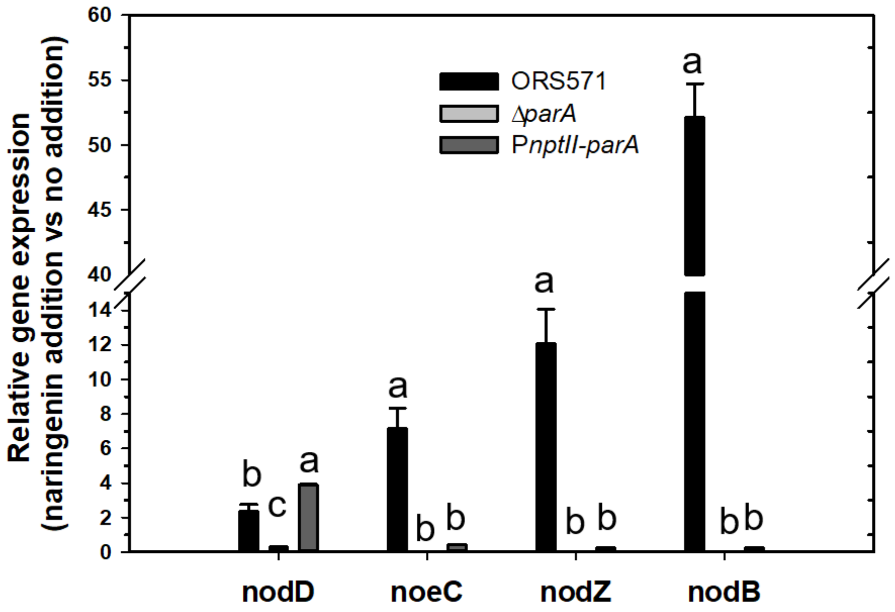

Figure S2. Differential expression of *nod* genes between free-living cells grown in L2+N medium with naringenin and L2+N medium alone.

The transcript levels of nodulation-related genes were determined by qPCR. The values are the means±standard deviations of three biological replicates (post hoc analysis: Tukey's HSD test).

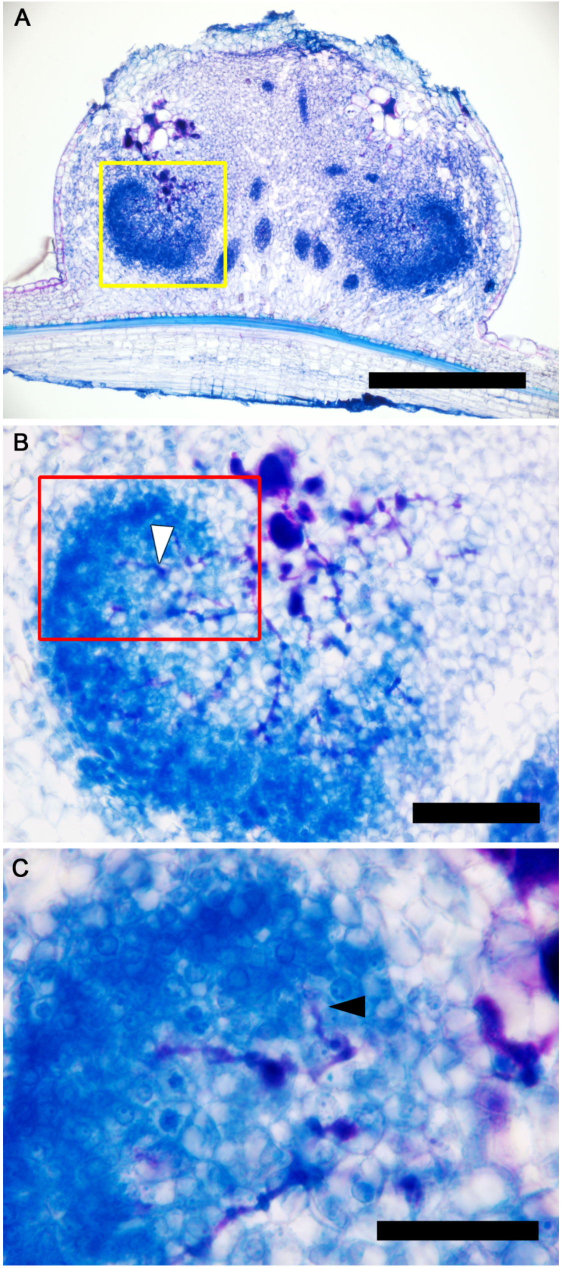

Figure S3. Reticular structure of P*nptII-parA* infection threads.

P*nptII-parA* stem-nodule samples were harvested at 7 dpi, fixed in FAA and sliced into 30 μm sections. The sections were stained with toluidine blue O. The yellow box in panel **A** and the red box in panel **B** indicate the fields of view of panels **B** and **C**, respectively. The white arrowhead indicates the reticular structure of the infection thread, and the black arrowhead indicates the infection thread, which was submerged in plant meristem cells. Scale bars indicate 500 μm, 100 μm, and 50 μm in panels A, B and C, respectively.

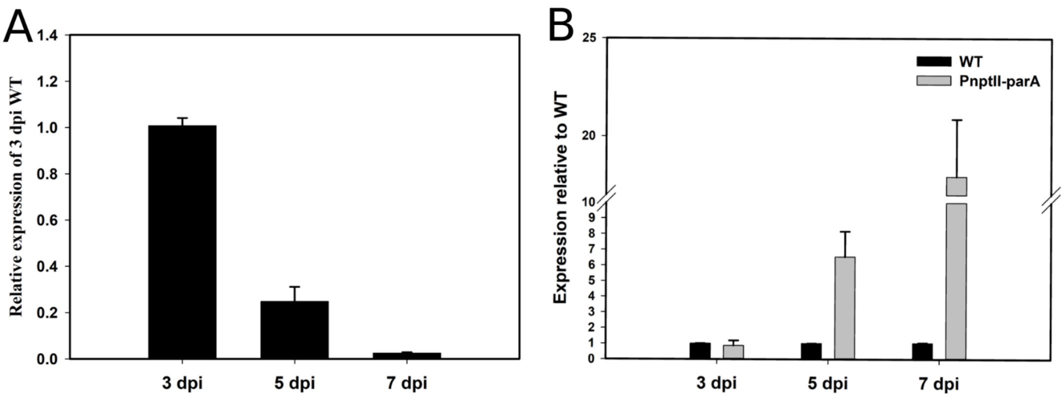

Figure S4. Relative gene expression of *SrGA20ox1*.

Expression of the plant gibberellin related genes *SrGA20ox1* during nodulation. *SrGA20ox1* expression pattern in ORS571-inoculated plants is shown as relative mRNA expression compared to 3 dpi ORS571 (A), and *SrGA20ox1* expression in P*nptII-parA* is shown as relative expression compared to ORS571 at the same time point (B). The values are the means±standard deviations of three biological replicates.

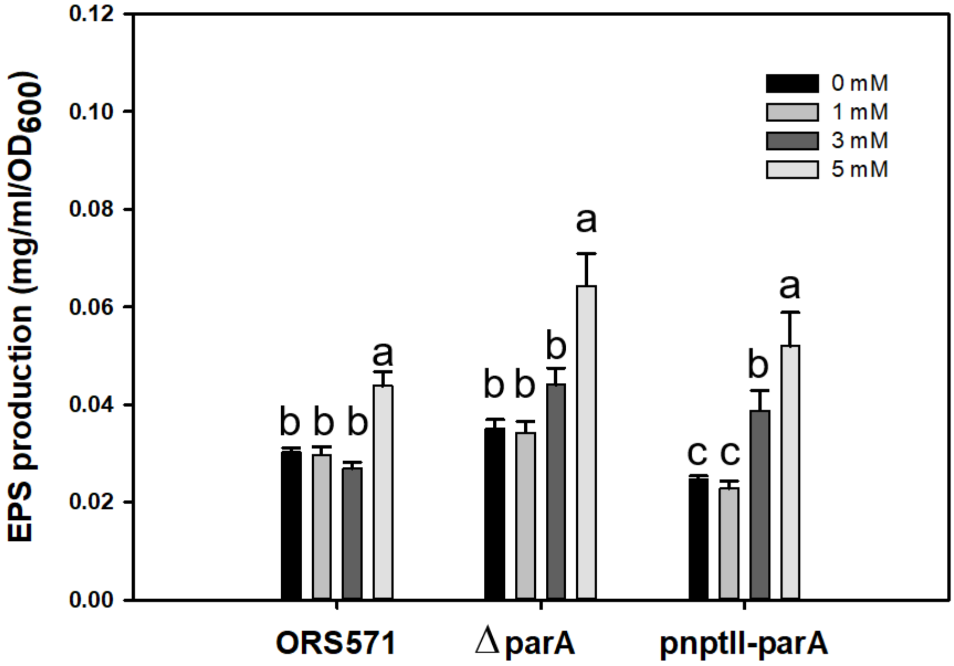

H_2O_2 concentration	Survival rate (%)		
0 mM	100	100	100
1 mM	100.17	99.09	99.44
3 mM	92.74	90.50	92.06
5 mM	86.69	86.35	88.45
	PI stained cell (%)		
0 mM	2.6	3	18.9
5 mM	9.3	23.4	25.1

Figure S5. EPS production, viability (survival rate) and cell membrane permeabilization assay of ORS571 derivative under H_2O_2 treatment.

The broth was treated with H_2O_2 for 30 min at a final concentration of 0, 1, 3, 5 mM. EPS from each strain was isolated and measured by the phenol-sulfuric acid method. The values are the means±standard deviations of three biological replicates. $P<0.05$ (post hoc analysis: Tukey). The viability (survival rate) of each bacterium under different treatments was measured by number of colony-forming units. The values was calculated from three biological replicates. The cell membrane permeability of the respective ORS571 derivative was measured by staining cells with propidium iodide (PI) dye. In each experiment, over 1,000 cells were analyzed.
